# Supplementary material for: HIVprotI: an integrated web based platform for prediction and design of HIV proteins inhibitors
Source: J Cheminform. 2018 Mar 9;10:12. doi: 10.1186/s13321-018-0266-y (PMC5845081; doi:10.1186/s13321-018-0266-y)
Supplement: Supplementary file 2 — Additional file 2. Source code of HIVProtI web server. [file 13321_2018_266_MOESM2_ESM.zip › HIVprotI_Source-code/hivprotI/weka-3-6-6/doc/weka/core/Memory.html]

Memory


---


|  |  |  |
| --- | --- | --- |
| **Overview** \| **Package** \| **Class** \| **Tree** \| **Deprecated** \| **Index** \| **Help** \| **Weka's home** | |  |
| **PREV CLASS**   **NEXT CLASS** | **FRAMES**    **NO FRAMES**     **All Classes** |  |
| SUMMARY: NESTED \| FIELD \| CONSTR \| METHOD | DETAIL: FIELD \| CONSTR \| METHOD |  |


---


## weka.core Class Memory

```
java.lang.Object
  weka.core.Memory
```

**All Implemented Interfaces:**: RevisionHandler

---

``` public class Memory extends java.lang.Object implements RevisionHandler ```

A little helper class for Memory management. Very crude, since JDK 1.4
doesn't offer real Memory Management.

The memory management can be disabled by using the setEnabled(boolean)
method.

**Version:**
:   $Revision: 1.7 $

**Author:**
:   FracPete (fracpete at waikato dot ac dot nz)

**See Also:**: `setEnabled(boolean)`

---

**Constructor Summary**

`Memory()`
  
          initializes the memory management without GUI support

`Memory(boolean useGUI)`
  
          initializes the memory management


| **Method Summary** | |
| --- | --- |
| `long` | `getCurrent()`             returns the current memory consumption |
| `long` | `getInitial()`             returns the initial size of the JVM |
| `long` | `getMax()`             returns the maximum amount of memory that can be assigned |
| `java.lang.String` | `getRevision()`             Returns the revision string. |
| `boolean` | `getUseGUI()`             whether to display a dialog in case of a problem (= TRUE) or just print on stderr (= FALSE) |
| `boolean` | `isEnabled()`             returns whether the memory management is enabled |
| `boolean` | `isOutOfMemory()`             checks if there's still enough memory left. |
| `static void` | `main(java.lang.String[] args)`             prints only some statistics |
| `void` | `setEnabled(boolean value)`             sets whether the memory management is enabled |
| `void` | `showOutOfMemory()`             prints an error message if OutOfMemory (and if GUI is present a dialog), otherwise nothing happens. |
| `void` | `stopThreads()`             stops all the current threads, to make a restart possible |
| `static double` | `toMegaByte(long bytes)`             returns the amount of bytes as MB |

**Methods inherited from class java.lang.Object**

`equals, getClass, hashCode, notify, notifyAll, toString, wait, wait, wait`

**Constructor Detail**

### Memory

```
public Memory()
```

:   initializes the memory management without GUI support

---


### Memory

```
public Memory(boolean useGUI)
```

:   initializes the memory management

    **Parameters:**: `useGUI` - whether a GUI is present


**Method Detail**

### isEnabled

```
public boolean isEnabled()
```

:   returns whether the memory management is enabled

    :   **Returns:**: true if enabled

---


### setEnabled

```
public void setEnabled(boolean value)
```

:   sets whether the memory management is enabled

    :   **Parameters:**: `value` - true if the management should be enabled

---


### getUseGUI

```
public boolean getUseGUI()
```

:   whether to display a dialog in case of a problem (= TRUE) or just print
    on stderr (= FALSE)

    :   **Returns:**: true if the GUI is used

---


### getInitial

```
public long getInitial()
```

:   returns the initial size of the JVM

    :   **Returns:**: the initial size in bytes

---


### getCurrent

```
public long getCurrent()
```

:   returns the current memory consumption

    :   **Returns:**: the current size in bytes

---


### getMax

```
public long getMax()
```

:   returns the maximum amount of memory that can be assigned

    :   **Returns:**: the maximum size in bytes

---


### isOutOfMemory

```
public boolean isOutOfMemory()
```

:   checks if there's still enough memory left. if ENABLED is true, then
    false is returned always

    :   **Returns:**: true if out of memory (only if management enabled, otherwise always false)

---


### toMegaByte

```
public static double toMegaByte(long bytes)
```

:   returns the amount of bytes as MB

    :   **Returns:**: the MB amount

---


### showOutOfMemory

```
public void showOutOfMemory()
```

:   prints an error message if OutOfMemory (and if GUI is present a dialog),
    otherwise nothing happens. isOutOfMemory() has to be called beforehand,
    since it sets all the memory parameters.

    :   **See Also:**: `isOutOfMemory()`, `m_Enabled`

---


### stopThreads

```
public void stopThreads()
```

:   stops all the current threads, to make a restart possible

---


### getRevision

```
public java.lang.String getRevision()
```

:   Returns the revision string.

    :   **Specified by:**: `getRevision` in interface `RevisionHandler`
    :   **Returns:**: the revision

---


### main

```
public static void main(java.lang.String[] args)
```

:   prints only some statistics

    :   **Parameters:**: `args` - the commandline arguments - ignored


---


|  |  |  |
| --- | --- | --- |
| **Overview** \| **Package** \| **Class** \| **Tree** \| **Deprecated** \| **Index** \| **Help** \| **Weka's home** | |  |
| **PREV CLASS**   **NEXT CLASS** | **FRAMES**    **NO FRAMES**     **All Classes** |  |
| SUMMARY: NESTED \| FIELD \| CONSTR \| METHOD | DETAIL: FIELD \| CONSTR \| METHOD |  |


---
